# Author Correction: A metal-free photocatalyst for highly efficient hydrogen peroxide photoproduction in real seawater

**DOI:** 10.1038/s41467-021-21490-z

**Published:** 2021-02-15

**Authors:** Qingyao Wu, Jingjing Cao, Xiao Wang, Yan Liu, Yajie Zhao, Hui Wang, Yang Liu, Hui Huang, Fan Liao, Mingwang Shao, Zhenghui Kang

**Affiliations:** 1 Institute of Functional Nano and Soft Materials Laboratory (FUNSOM), Jiangsu Key Laboratory for Carbon-Based Functional Materials & Devices, Soochow University, 215123 Suzhou, PR China; 2 Macao Institute of Materials Science and Engineering, Macau University of Science and Technology, Taipa, 999078 Macau SAR China

**Keywords:** Photocatalysis, Photocatalysis, Quantum dots

Correction to: *Nature Communications* 10.1038/s41467-020-20823-8, published online 20 January 2021.

The original version of this Article contained an error in Fig. 5d, in which the label ‘·O_2_^-^’ in the upper left corner of Fig. 5, panel d should be replaced by ‘DMPO-·O_2_^-^’. This has been corrected in both the PDF and HTML versions of the Article.

The original version of the Supplementary information associated with this Article contained an error in Supplementary Figure S26, in which the label ‘·OH’ in the upper left corner of this figure should be replaced by ‘DMPO-·OH’. The HTML has been updated to include a corrected version of the Supplementary information.

The original version of this Article contained an error in the second sentence of the fourth paragraph of the ‘Proposed photocatalytic mechanism’ subsection of the ‘Results’ section, which incorrectly read ‘As shown in Fig. S26, there is no signal of free ·OH radicals is observed under darkness and illumination, confirming that no free ·OH radicals are produced in photocatalytic reaction.’ The correct version replaces this sentence with ‘As shown in Figure S26, there is no signal of DMPO-·OH observed under darkness and illumination, confirming that no ·OH are produced in photocatalytic reaction’. This has been corrected in both the PDF and HTML versions of the Article.

The original version of this Article contained an error in the fourth sentence of the fourth paragraph of the ‘Proposed photocatalytic mechanism’ subsection of the ‘Results’ section, which incorrectly read ‘Notably, four mainly ·O_2_^−^ signals are observed after light illumination in Fig. 5d, suggesting that ·O_2_^−^ is the main free radical in the photocatalytic reaction.’ The correct version replaces this sentence with ‘Notably, six DMPO-·O_2_^-^ signals are observed after light illumination in Fig. 5d, suggesting that ·O_2_^-^ is the main free radical in the photocatalytic reaction’. This has been corrected in both the PDF and HTML versions of the Article.

The original version of the Supplementary information associated with this Article contained an error in the final sentence of the first paragraph in the ‘1.1 Characterization’ subsection of the ‘1. Supplemental Experimental Procedures’ section, which incorrectly read ‘The free radicals produced in photocatalytic reaction were detected by electron paramagnetic resonance (EPR, BrookerA300).’ The correct version states ‘With the addition of DMPO, the free radicals produced in photocatalytic reaction were detected by electron paramagnetic resonance (EPR, BrookerA300) at the microwave frequencies of 9.8 GHz.’ in place of ‘The free radicals produced in photocatalytic reaction were detected by electron paramagnetic resonance (EPR, BrookerA300).’ The HTML has been updated to include a corrected version of the Supplementary information.

The original version of this Article contained an error in the author affiliations. Affiliation two incorrectly read ‘Macau Institute of Materials Science and Engineering, Macau University of Science and Technology, Macau, China'. The correct version states ‘Macao Institute of Materials Science and Engineering, Macau University of Science and Technology, Taipa 999078, Macau SAR, China’ in place of ‘Macau Institute of Materials Science and Engineering, Macau University of Science and Technology, Macau, China'.

This has now been corrected in both the PDF and HTML versions of the Article.

## Supplementary information

Supplementary materials

