# Supplementary material for: A metal-free photocatalyst for highly efficient hydrogen peroxide photoproduction in real seawater
Source: Nat Commun. 2021 Jan 20;12:483. doi: 10.1038/s41467-020-20823-8 (PMC7817682; doi:10.1038/s41467-020-20823-8)
Supplement: Supplementary file 1 — Supplementary Information [file 41467_2020_20823_MOESM1_ESM.docx]

**Supplementary materials**

A metal-free photocatalyst for highly efficient hydrogen peroxide photoproduction in real seawater

Qingyao Wu^1^, Jingjing Cao^1^, Xiao Wang^1^, Yan Liu^1^, Yajie Zhao^1^, Hui Wang^1^, Yang Liu^1^*, Hui Huang^1^, Fan Liao^1^, Mingwang Shao^1^* and Zhenghui Kang^1,2^*

^1^*Institute of Functional Nano and Soft Materials Laboratory (FUNSOM), Jiangsu Key Laboratory for Carbon-Based Functional Materials & Devices, Soochow University, Suzhou 215123, PR China.*

^2^*Macao Institute of Materials Science and Engineering, Macau University of Science and Technology, Taipa 999078, Macau SAR, China.*

*Correspondence: [yangl@suda.edu.cn;](mailto:yangl@suda.edu.cn;) mwshao@suda.edu.cn; zhakng@suda.edu.cn

**This PDF file includes:**

Supplemental experimental section

Supplemental computational details and methods

Supplemental thermodynamic-kinetic model

Supplemental Figures.

Supplemental tables.

Figures S1 to S28

Table S1 to S4

Supplemental references

**1. Supplemental Experimental Procedures**

**1.1 Characterization**

The crystal structure of the as-prepared products was characterized by power X-ray diffraction (XRD) through a PIXcel3D X-ray diffractometer (Empyrean, Holland Panalytical) equipped with Cu Kα radiation (*λ* = 0.154 nm). The surface morphology of the samples was observed through a scanning electron microscope (SEM) and transmission electron microscopy (TEM) equipped with a FEI-Tecnai F20 transmission electron microscope (200 kV accelerating voltage). The particle size distribution of the catalysts was determined by dynamic light scattering (DLS). The Fourier transform infrared (FT-IR) spectra were recorded on a Hyperion spectrophotometer (Bruker) at the scan range of 400-4000 cm^-1^. With the addition of DMPO, the free radicals produced in photocatalytic reaction were detected by electron paramagnetic resonance (EPR, BrookerA300) at the microwave frequencies of 9.8 GHz.

Ultraviolet-visible (UV-vis) absorption spectra were acquired on a UV/VIS/NIR spectrophotometer (Lambda 750, Perkinelmer). The valence band of the photocatalyst was obtained by ultraviolet photoelectron spectroscopy (UPS) with He I (21.22 eV) as the monochromatic light source and a total instrumental energy resolution of 100 meV.

Photoelectrochemical tests were performed on the CHI 920C workstation using a three-electrode system, in which the carbon rod was used as the counter electrode, the saturated calomel electrode (SCE) as the reference electrode, and the glassy carbon (GC) electrode as working electrode. The photo-responsive property of the photocatalysts was characterized by a time-current (*i-t*) curve at open circuit potential (OCP) in seawater with a light-emitting diode (LED, 100 W) lamp as the light source. Electrochemical impedance spectra (EIS) measurements were carried out in seawater in the range of 1 MHz to 0.01 Hz and alternating current voltage amplitude of 5 mV.

Transient photovoltage (TPV) was measured on a home-made system, which was excited by a nanosecond laser radiation pulse (wavelength of 355 nm and the repetition rate is 5 Hz) from a third harmonic Nd:YAG (Beamtech Optronics Co., Ltd). In the test, 5 mg sample was covered on the Pt network (1 cm × 1 cm) as working electrodes and the signal of the TPV was amplified by the amplifier and recorded by the oscilloscope. The schematic diagram of this test system was shown in Figure S4.

The *in-situ* TPV test was conducted on the sample film (1 mg, 1 cm × 1.5 cm), which was deposited on indium-tin oxide (ITO) glass substrate. The modified ITO was used as the working electrode and the Pt wire as the counter electrode. During the measurement, the sample film was respectively wetted with N_2_-saturated, O_2_-saturated acetonitrile, 0.1 vol% N_2_-saturated acetonitrile/water solution and 0.1 vol% N_2_-saturated acetonitrile/seawater solution (v/v). All the measurements were performed at room temperature and under ambient pressure.

**1.2. Electron transfer number (*n*)**

The electron transfer number for oxygen reduction reaction was performed on a rotating disk electrode (RDE) in O_2_-saturated seawater with different rotating speed. The average number of electrons (*n*) can be estimated by linear regression of the plots using the following equations:^1^

$\frac{1}{j}\text{ = }\frac{1}{j_{k}}+\frac{1}{B}\times\frac{1}{\sqrt{\omega}}$ (1)

$B=0.2nF\nu^{-1/6}CD^{2/3}$ (2)

Here, *j* is the current density, *j*_k_ is the kinetic current density, *ω* is the rotating speed (rpm), *F* is the Faraday constant (96485 C mol^-1^), *ν* is the kinetic viscosity of water (0.01 cm^2^ s^-1^), *C* is the bulk concentration of O_2_ in water (1.26 × 10^-3^ mol cm^-3^), and *D* is the diffusion coefficient of O_2_ (2.7 × 10^-5^ cm^2^ s^-1^).

The electron transfer number of water oxidation reaction was characterized by rotating disk-ring electrodes (RRDE) testing system (RRDE-3, ALS Co., Ltd). In N_2_-saturated seawater, the *i-t* curve was tested at the scan rate of 10 mV s^-1^ with the rotating speed of 1600 rpm. The disk potential was set at OCP to avoid water oxidation during photochemical and electrochemical catalysis process. The ring potential was kept at 0.9 V *vs*. SCE, which can oxidize the generated H_2_O_2_ from the disk into O_2_. Finally, n is obtained by the following formula:^2^

$\text{n}\text{=}\frac{\text{4}{\text{∆}\text{I}}_{d}}{{\Delta I}_{d}+\frac{\Delta I_{r}}{N}}$ (3)

Here, *I*_d_ represents the disk current and *I*_r_ represents the ring current, while *N* is the RRDE collection efficiency determined to be 0.40.

**1.3** **Cyclic voltammetry (CV) measurement**

The cyclic voltammetry (CV) method was carried by a standard three-electrode system with CHI 760E workstation. The sample-modified (PM-CDs-0 and PM-CDs-30) glassy carbon (GC), Ag/AgCl electrode and carbon electrode were used as the work electrode, reference electrode and counter electrode, respectively. In this experiment, 4 μL catalyst solution (2 mg mL^-1^) and 5 μL Nafion solution (0.5 wt%) were dropped onto the working area of a cleaned GC electrode and put naturally to dry. The CV curves were measured in N_2_-saturated 0.1 M BMIMPF_6_ solution with a scan rate of 50 mV s^-1^. Ferrocene was added into the above solution as an internal standard with a concentration of 1 mg mL^-1^. The energy levels of the catalyst were calculated from the onset oxidation ($E_{\mathrm{onset}}^{\mathrm{ox}}$), reduction ($E_{\mathrm{onset}}^{\mathrm{red}}$) potential and the onset oxidation potential of ferrocene ($E_{\mathrm{ferrocene}}$).

**1.4 Apparent quantum yield (AQY)**

To evaluate the apparent quantum yield (AQY) of PM-CDs-30, 10 mg photocatalyst and 20 mL seawater was put into a quartz photo-reactor vial with a diameter of 32 mm. Afterwards, the system was constantly stirred for 5 h under light irradiation with a band-pass filter (*λ*_0_ = 380, 460, 530 or 630 nm). KMnO_4_ (0.1 M) was used to calculate the amount of H_2_O_2_ generated.

The average intensity of irradiation was measured by ILT 950 Spectroradiometer (International Light Technologies), and the detail information was displayed in Table S3. The irradiation area was calculated to be 8.04 cm^2^. The number of incident photons (M) is calculated by the following equation:^3^

$M=\frac{E\lambda}{hc}$ (4)

In the equation, *E*, *λ*, *h* and *c* are the average intensity of irradiation, the wavelength of the irradiation, Planck constant and the speed of light, respectively.

The quantum efficiency was calculated from the following equation: ^4^

$\text{AQE=}\frac{2\times\text{ number of evolved }\text{H}_{2}O_{2}\text{ molecules}}{\text{number of incident photons}}\times100\%$ (5)

**1.5 Solar-to-chemical conversion (SCC) efficiency**

In the experiment, the multichannel photochemical reaction system equipped with the visible light (*λ* ≥ 420 nm) as the light source and PM-CDs-30 (10 mg) as the catalyst was used to calculated the solar-to-chemical conversion (SCC) efficiency. After 5 h of illumination, the total incident power over the 8.04 cm^2^ irradiation area was 34.8 mW cm^-2^. So that the total input energy in 5 h was:

*E*_soalr_=5036.2 J

During the photocatalytic reaction, 88.8 μmol H_2_O_2_ was detected, which indicated that the energy of produced hydrogen peroxide was:

$$E_{H_{2}O_{2}}=n\left( H_{2}O_{2} \right)\times\Delta G\left( H_{2}O_{2} \right)=88.8\times{10}^{-6}\times117{\times10}^{3} J=10.4 J$$

The SCC conversion efficiency of PM-CDs-30 was determined to be:

$$SCC=\frac{E_{H_{2}O_{2}}}{E_{\mathrm{solar}}}\times100\%=\frac{10.4 J}{5036.2 J}\times100\%=0.21\%$$

**1.6** **Electrochemical measurement of the PM-CDs-30 for decomposition of H_2_O_2_**

The H_2_O_2_ decomposition behavior of PM-CDs-30 was measured by cycle voltammetry (CV) in 0.2 M (pH = 7) phosphate buffered solution with 25 mM H_2_O_2_. A standard three-electrode system with CHI 760E workstation was used. The carbon electrode and the saturated calomel electrode were used as the counter electrode and the reference electrode, respectively. The bare glassy carbon (GC) electrode (3 mm diameter) and PM-CDs-30-modified GC were used as the work electrodes, which are used as the control group and experiment group respectively. Here, 4 μL catalyst solution (2 mg mL^-1^) and 5 μL of 0.5 wt % Nafion solution were dropped onto the working area of a cleaned GC electrode and put naturally to dry. The CV curves were measured under darkness with a scan rate of 50 mV s^-1^ and the results are shown in Figure S18a.

**1.7 Degradation of H_2_O_2_ by PM-CDs-30**

10 mg PM-CDs-30 was dispersed in 20 mL water with 50 μmol H_2_O_2_ added. The reaction vessels were then placed under dark conditions and stirred. The content of H_2_O_2_ in the solution was detected after 0, 6, 12, and 24h. The change of H_2_O_2_ content is shown in Figure S18b.

**1.8 The salting out experiments**

10 mg PM-CDs-30 was added to ultrapure water (50mL), seawater (50mL), 2.5 mol L^-1^ NaCl solution (50mL) and 5 mol L^-1^ NaCl solution (50mL). The mixture was then ultrasonic for 10 min to disperse evenly. Next, the mixed solution was left to stand, and the upper solution was taken at intervals (0, 1, 1, 5, 8, 12, and 24 h) for UV-vis test to determine the content of catalyst in the solution. The results are shown in Figure S19.

**1.9 Electrochemical oxygen evolution reaction tests**

The electrochemical oxygen evolution reaction experiment was performed by CHI 920C electrochemical workstation with a three-electrode system. The working electrode was a modified glassy carbon electrode (GCE, 3 mm in diameter). A saturated calomel electrode (SCE) was employed as the reference electrode and a carbon rod as the counter electrode. To prepare the working electrode, 3 mg PM-CDs-30 was dispersed in 1 mL Nafion solution including (0.5 wt%) to form a homogeneous suspension. Then, 5 µL of the suspension was loaded on the GCE. The catalyst was tested in 0.1 M Na_2_SO_4_ solution and seawater solution at the scanning speed of 50 mV s^-1^.

**1.10 Electrochemical oxygen reduction reaction tests**

The electrochemical oxygen reduction reaction experiment was performed by using a CHI 920C electrochemical workstation with a three-electrode system. The working electrode was a modified glassy carbon electrode (GCE, 3 mm in diameter). A saturated calomel electrode (SCE) was used as the reference electrode and a carbon rod as the counter electrode. To prepare the working electrode, 3 mg PM-CDs-30 was dispersed in 1 mL Nafion solution including (0.5 wt%) to form a homogeneous suspension. Then, 5 µL of the suspension was loaded on the GCE. The catalyst was tested in O_2_-saturated 0.1 M Na_2_SO_4_ solution and O_2_-saturated seawater solution at the scanning speed of 50 mV s^-1^.

**2. Supplemental computational details and methods**

The Vienna Ab initio Simulation Package (VASP) were applied to perform the spin-polarized density functional theory (DFT) calculations. The electron−ion interactions were described by the projector augmented wave (PAW) method proposed by Blöchl and implemented by Kresse. The electronic ground states were treated with the Perdew-Burke-Ernzerhof (PBE) within the generalized gradient approximation (GGA) exchange correlations potentials. The cut-off energy of plane wave basis was set as 400 eV, and the van der Waals interactions were described with the vdW-D3 method. The graphene ribbon was modeled with a 4 × 4 unit cell in the type of zigzag in which including 32 carbon atoms. For the Brillouin zone sampling, a 3 × 3 K point mesh was used. Vacuum region of 22 Å and 36 Å were applied separately along two directions to avoid the interactions between transnationally periodic images. During the structure optimization, all the atoms in the cell were allowed to relax. The optimization was stopped when the force residue on the atom was smaller than 0.02 eV/Å. The climbing image nudged elastic band (CI-NEB) method with six images was applied for searching the minimum energy paths of all reactions and finding the transition states. The transition states were then picked as the input structures of the subsequent dimer calculations.

**3. Supplemental** **Thermodynamic-Kinetic Model**

**3.1 Oxygen evolution reaction (OER)**

For photocatalytic OER (O_2_ production), we first carried out its electrocatalytic reaction to obtain its hole transfer number (*n*_1_), concentration index (*m*), reaction activation energy (*E*_1_), and reaction initiation potential (*U*_1_).

Then, the following formula could be obtained.

$J_{f1}=n_{1}{Fk}_{10}\exp\left( \frac{{-E}_{1}}{RT} \right)\exp\left( \frac{\alpha F\left( U_{1}-U_{10} \right)}{RT} \right)C_{1}^{m}$ (6)

Here, *J*_f1_ is the current; *n*_1_ is the number of hole transfer (*n*_1_ = 4); *F* is Faraday constant (*F* = 96485 C mol^-1^); *k*_10_ is the rate constant of OER; *E*_1_ is activation energy of OER; *R* is the gas constant (*R* = 8.314 J mol^-1^K^-1^); *T* is reaction temperature (*T* = 298.15 K); *α* is a constant (*α* = 0.5); *U*_1_ is a potential (*vs.* RHE) in Tafel region; *U*_10_ is the potential of water oxidation (*U*_10_ = 1.23 V); *C*_1_^m^ is the concentration of hydroxyl of water (m = 1).

Therefore, formula 6 could be simplified as follows:

$J_{f1}=k_{10}^{'}\exp\left( \frac{\alpha F\left( U_{1}-U_{10} \right)}{RT} \right)$ (7)

A potential-current pair (*U*_1_, *J*_f1_) in the Tafel region of the OER curve (measured in seawater) was selected (Figure S14a) and substituted into formula 7 to obtain *k*_10_*’*.

Then, the photocatalytic reaction rate was calculated. We Substituted *k*_10_*’*, *U*_1_ (the valence band, 1.54 V), and *U*_10_ (1.23 V) into equation 7 to obtain *J*_1_.

**3.2 Oxygen reduction reaction (ORR)**

For photocatalytic ORR (H_2_O_2_ production), we also first performed its electrocatalytic reaction to obtain its hole transfer number (*n*_2_), concentration index (*n*), reaction activation energy (*E*_2_), and reaction initiation potential (*U*_2_).

Thus, the following formula could be obtained.

$J_{f2}=n_{2}{Fk}_{20}\exp\left( \frac{{-E}_{2}}{RT} \right)\exp\left( \frac{\alpha F\left( U_{2}-U_{20} \right)}{RT} \right)C_{2}^{n}$ (8)

In this equation, *J*_f1_ is the current; *n*_2_ is the number of hole transfer (*n*_2_ = 2); *F* is Faraday constant (*F* = 96485 C mol^-1^); *k*_20_ is the rate constant of ORR; *E*_2_ is activation energy of ORR; *R* is the gas constant (*R* = 8.314 J mol^-1^K^-1^); *T* is reaction temperature (*T* = 298.15 K); *α* is a constant (*α* = 0.5); *U*_2_ is a potential (*vs.* RHE) in Tafel region; *U*_20_ is the potential of water oxidation (*U*_20_ = 0.68 V); *C*_2_^n^ is the partial pressure of oxygen (n = 1).

Therefore, formula 8 could be simplified as follows:

$J_{f1}=k_{20}^{'}\exp\left( \frac{\alpha F\left( U_{2}-U_{20} \right)}{RT} \right){Po}_{2}$ (9)

Similarly, a potential-current pair (*U*_2_, *J*_f2_) in the Tafel region of the ORR curve (measured in seawater) was selected (Figure S14b) and substituted into formula 9 to obtain *k*_20_*’*.

Then, the photocatalytic reaction rate was calculated. We substituted *k*_20_*’*, *U*_2_ (the valence band, -0.4 V), and *U*_20_ (0.68 V) into formula 9, and get *J*_2_.

**3.3 Consumed charge ratio of OER/ORR in photocatalytic reaction**

A laser (355 nm) was used to excite the photocatalyst. A working electrode was prepared with PM-CDs-30 and placed into N_2_-saturated anhydrous acetonitrile to obtain the curve of current change over time (*I*_0_ = *I*_0_(*t*), no photocatalytic reaction). In addition, TPV curves of different photocatalytic reactions were obtained when seawater (vol% = 0.1%) and oxygen (saturation) were added as sacrificial agents to anhydrous acetonitrile, respectively.

These TPV curves were integrated (namely Q_0_, Q_1s_, Q_2_, respectively), and then the number of electrons consumed by the different reactions was obtained (Figure S15).

Among them, Q_0_ is the total charge generated by excitation in pure acetonitrile and eventually consumed by the whole circuit; Q_1s_ is the total electrons generated by excitation in 0.1 vol% seawater/acetonitrile solution after the holes consumed by water oxygen reaction; Q_2_ is the total electrons generated by excitation in O_2_-saturated acetonitrile solution after the electrons consumed by the oxygen reduction reaction.

Then, we could get the coefficient λ.

$\lambda=\frac{\mathrm{OER}}{\mathrm{ORR}}=\frac{1}{2}\times\frac{Q_{1s}-Q_{0}}{Q_{0}-Q_{2}}$ (10)

**2.4 Calculation of the reaction rate**

First, the effect of water oxidation on the rate was considered.

$$r_{1}=\lambda n_{1}Fk_{10}\exp\left( \frac{-E_{1}}{RT} \right)\exp\left( \frac{\alpha F\left( U_{1}-U_{10} \right)}{RT} \right)C_{1}^{m}\text{=}{\lambda k}_{10}^{'}\exp\left( \frac{aF\left( U_{1}-U_{10} \right)}{RT} \right)$$

Then, the effect of oxygen reduction in the rate was considered. Note that the rotational speed here was greater than zero (*ω* > 0).

$$r_{2}=n_{2}Fk_{20}\exp\left( \frac{-E_{2}}{RT} \right)\exp\left( \frac{\alpha F\left( U_{2}-U_{20} \right)}{RT} \right)C_{2}^{n}$$

$$=k_{20}^{'}\exp\left( \frac{\alpha F\left( U_{2}-U_{20} \right)}{RT} \right)\times{Po}_{2}\times\left( \frac{\omega}{1600} \right)^{0.5} (\omega>0)$$

Next, the effect of oxygen diffuses on the rate was considered. Note that the rotational speed here was greater than zero (*ω* > 0).

$$r_{3}=J_{\max}\left( 60 rpm \right)\times\left( \frac{\omega}{60} \right)^{0.8}\times Po_{2}$$

$$=J_{\max}\left( 1600 rpm \right)\times\left( \frac{60}{1600} \right)^{0.8}\times\left( \frac{\omega}{60} \right)^{0.8}\times Po_{2} \left( \omega>0 \right)$$

Last, the rate-limiting steps under different conditions, whose reaction rate was the reaction rate of PM-CDs-30, was obtained. Note that the rotational speed here was greater than zero (*ω* > 0).

$$r=min\left\{ r_{1},r_{2},r_{3} \right\}$$

$$=min\left\{ \lambda n_{1}Fk_{10}\exp\left( \frac{-E_{1}}{RT} \right)\exp\left( \frac{\alpha F\left( U_{1}-U_{10} \right)}{RT} \right)C_{1}^{m},n_{2}Fk_{20}\exp\left( \frac{-E_{2}}{RT} \right)\exp\left( \frac{\alpha F\left( U_{2}-U_{20} \right)}{RT} \right)C_{2}^{n},J_{\max}\left( 60 rpm \right)\times\left( \frac{\omega}{60} \right)^{0.8}\times Po_{2} \right\}$$

$$=\min\left\{ {\lambda k}_{10}^{'}\exp\left( \frac{aF\left( U_{1}-U_{10} \right)}{RT} \right),k_{20}^{'}\exp\left( \frac{\alpha F\left( U_{2}-U_{20} \right)}{RT} \right)\times{Po}_{2}\times\left( \frac{\omega}{1600} \right)^{0.5},J_{\max}\left( 1600 rpm \right)\times\left( \frac{60}{1600} \right)^{0.8}\times\left( \frac{\omega}{60} \right)^{0.8}\times Po_{2} \right\}$$

$$=\left\{ \text{8.97}\times\text{1}\text{0}^{4},\text{2.31}\times\text{1}\text{0}^{\text{11}}\times Po_{2}\times\left( \frac{\omega}{1600} \right)^{0.5},5.24\times{10}^{3}\times{(\frac{\omega}{60})}^{0.8}\times{Po}_{2} \right\} \left( \omega>0 \right)$$

Finally, the thermodynamic-kinetic model of PM-CDs-30 in pure water was also investigated according to the above way.

**4. Supplemental Figures**


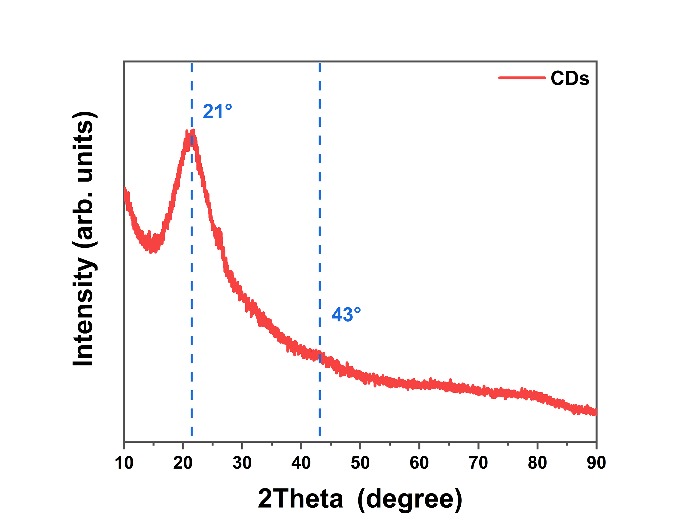


**Figure S1.** XRD pattern of CDs.


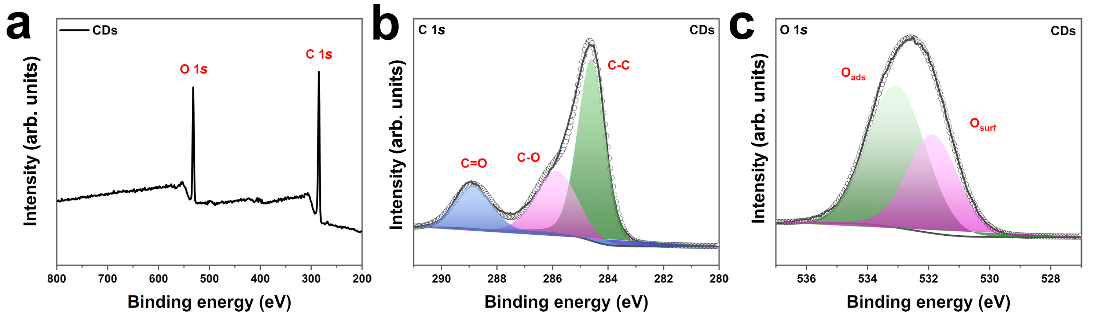


**Figure S2.** XPS spectra of CDs. (a) Full spectrum. (b) C 1*s* spectrum. (c) O 1*s* spectrum.


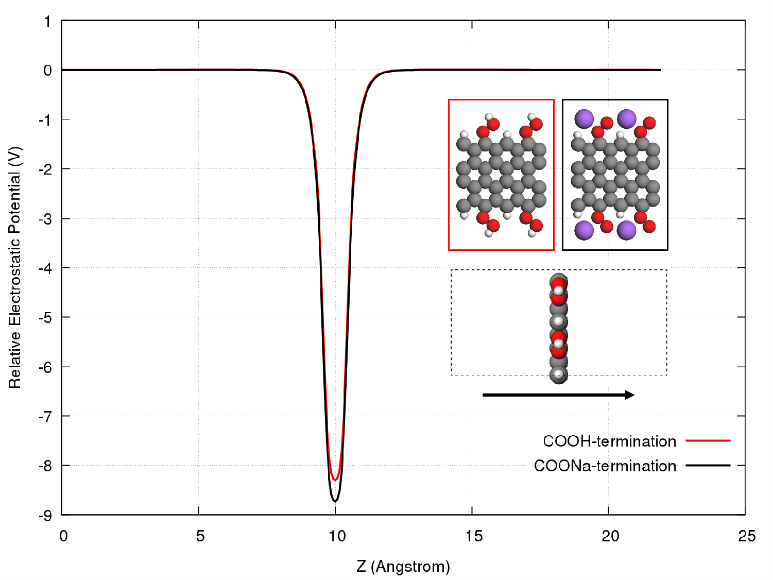


**Figure S3.** The plane-averaged potentials of nanoribbons plotted along the direction that perpendicular to the surface. The atomic configurations of carboxyl (-COOH) and sodium carboxylate (-COONa) graphene nanoribbons are shown in the inset image.


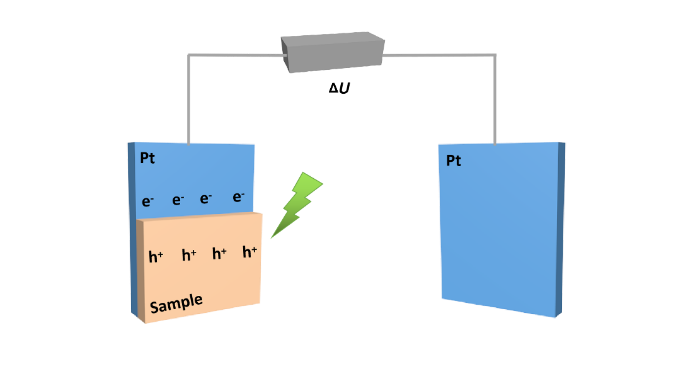


**Figure S4**. The schematic diagram of TPV test system.


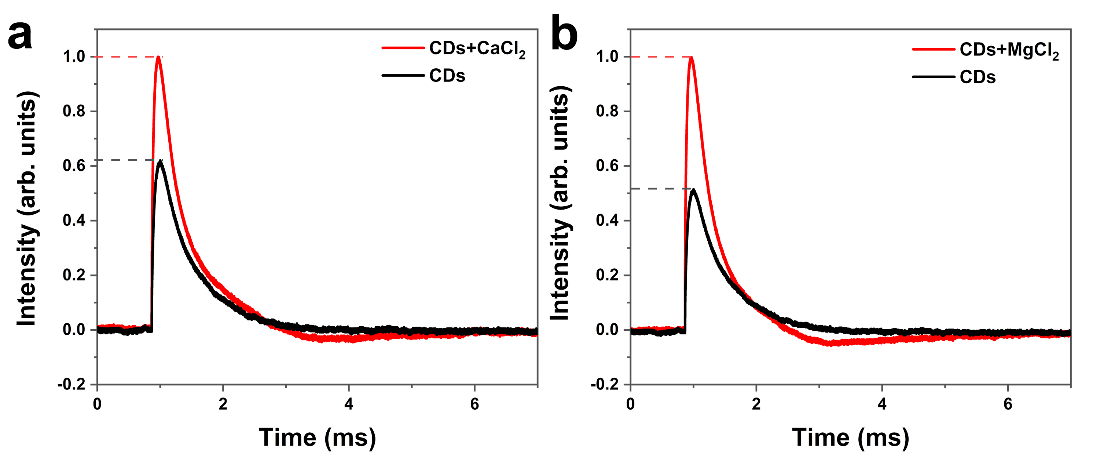


**Figure S5.** (a) TPV curves of CDs before and after adding MgCl_2_. (b) TPV curves of CDs before and after adding CaCl_2_.


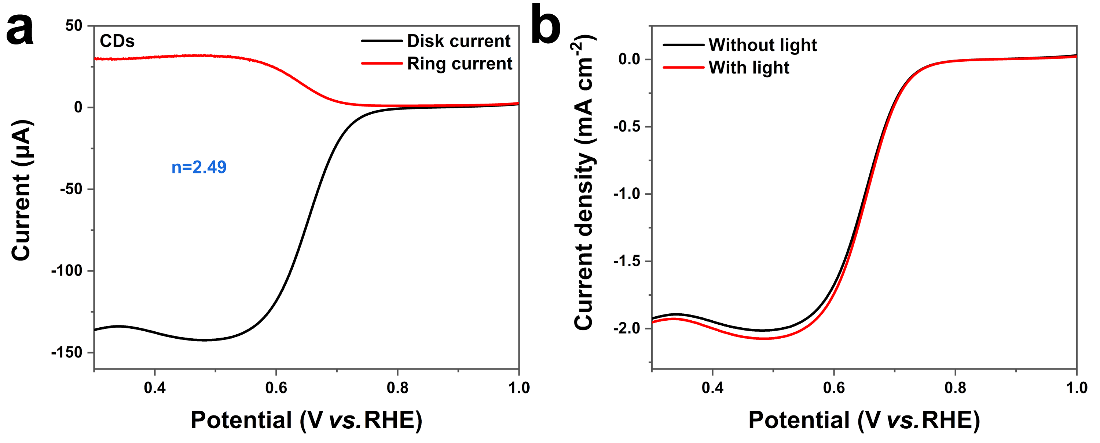


**Figure S6.** (a) Linear sweep voltammetry (LSV) curve of CDs-loaded electrode toward oxygen reduction reaction (ORR). (b) LSV curves of CDs-loaded electrode toward ORR with and without light.


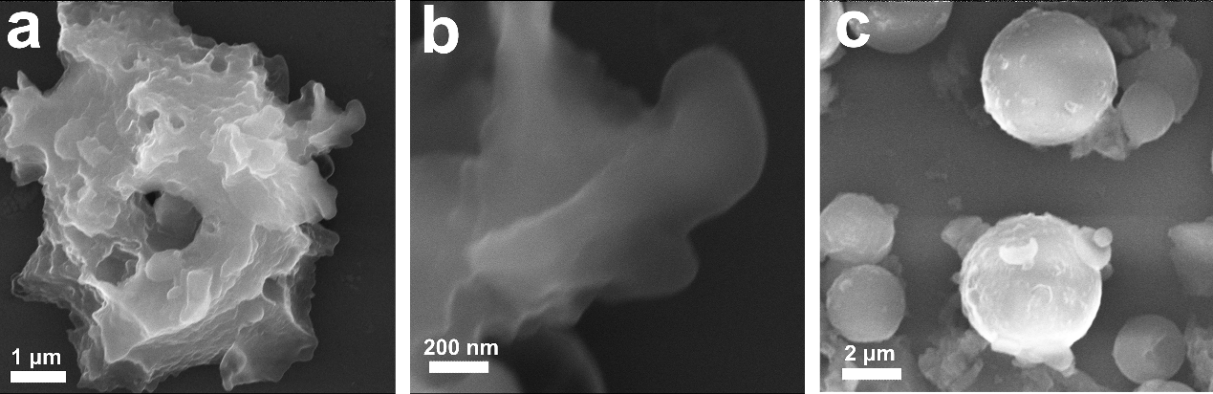


**Figure S7.** (a) SEM image of PM-CDs-30. (b) Local enlarged view of Figure S7a. (c) SEM image of PM-CDs-0.


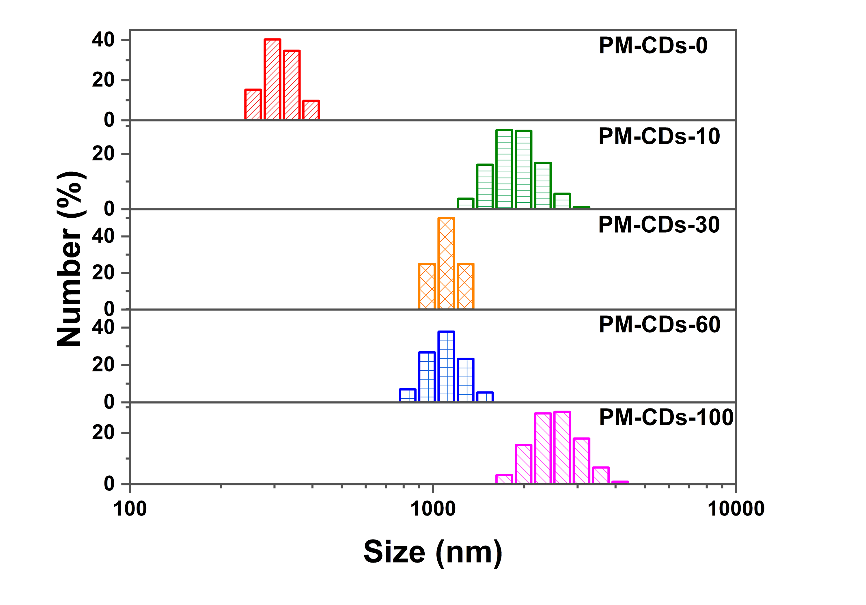


**Figure S8.** Particle size distribution diagram of PM-CDs-x.


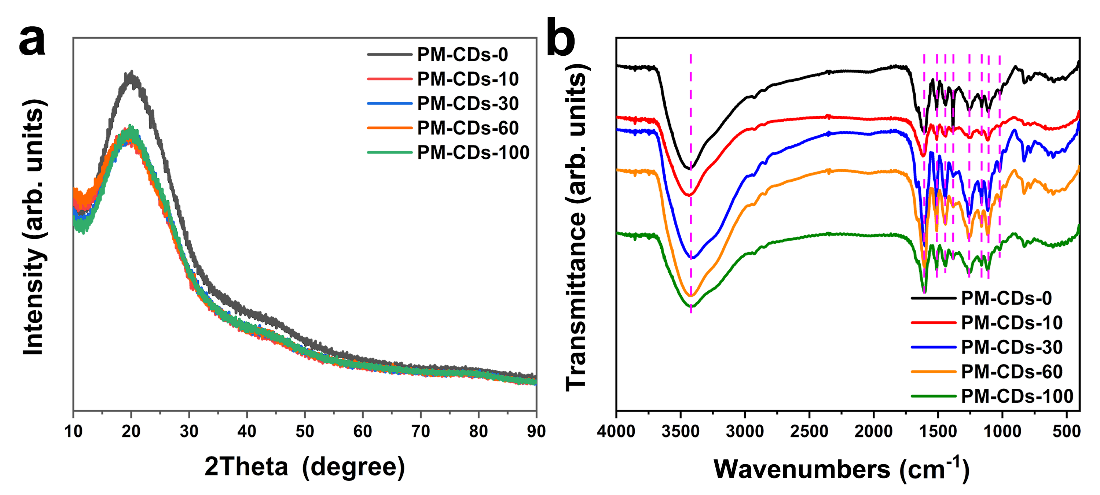


**Figure S9.** (a) XRD patterns of PM-CDs-x. (b) FT-IR spectra of PM-CDs-x.


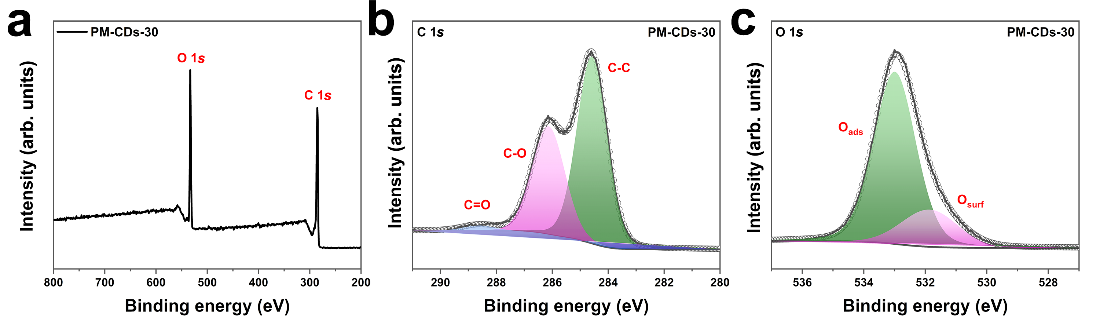


**Figure S10.** XPS spectra of PM-CDs-30. (a) Full spectrum. (b) C 1*s* spectrum. (c) O 1*s* spectrum.


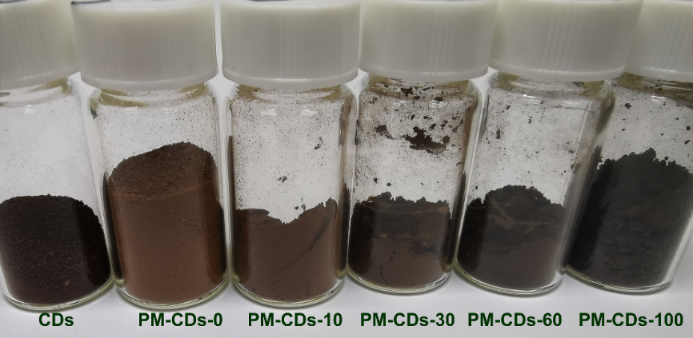


**Figure S11.** Pictures of different samples.


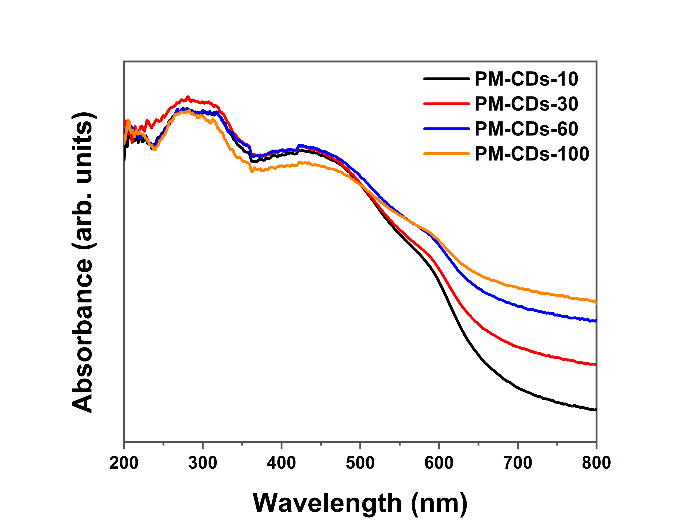


**Figure S12.** UV-vis absorption spectra of PM-CDs-x. Source data are provided as a Source Data file.


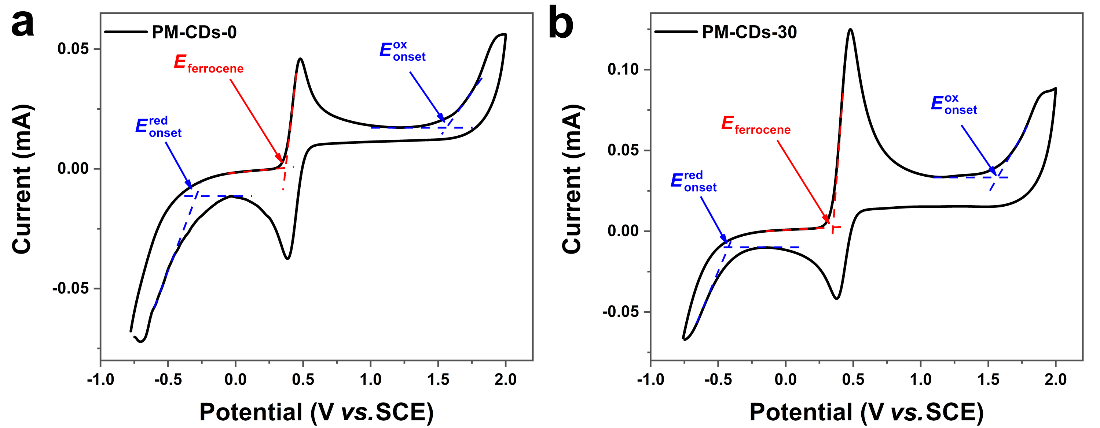


**Figure S13.** (a) CV curve of PM-CDs-0-modified glassy carbon (GC) electron in N_2_-saturated anhydrous acetonitrile (0.1 M BMIMPF_6_) with ferrocene as the internal standard. (b) CV curve of PM-CDs-30-modified GC electron in N_2_-saturated anhydrous acetonitrile (0.1 M BMIMPF_6_) with ferrocene as the internal standard. Source data are provided as a Source Data file.


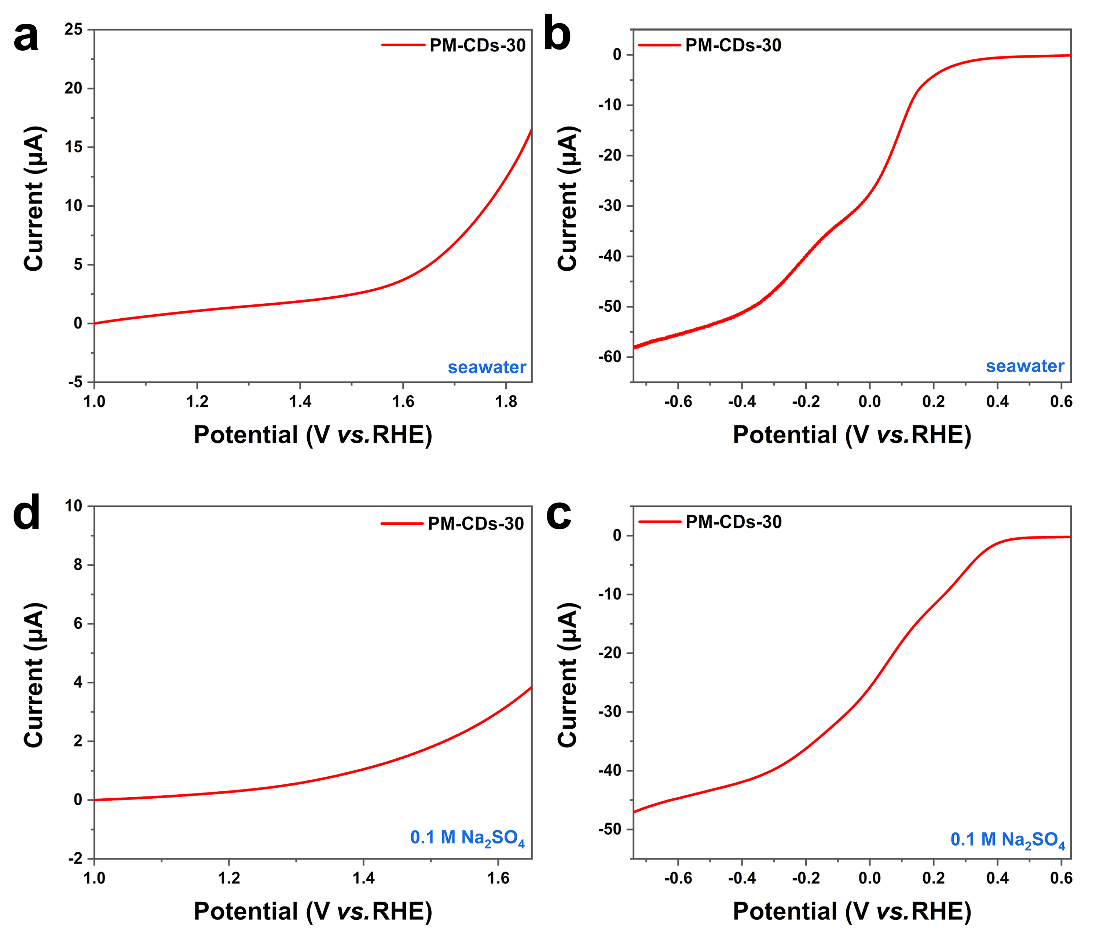


**Figure S14.** Electrocatalytic tests of PM-CDs-30. (a) LSV curve towards oxygen evolution reaction (OER) in seawater. (b) LSV curve towards ORR in seawater. (c) LSV curve towards OER in 0.1 M Na_2_SO_4_. (d) LSV curve towards ORR in 0.1 M Na_2_SO_4_. Source data are provided as a Source Data file.


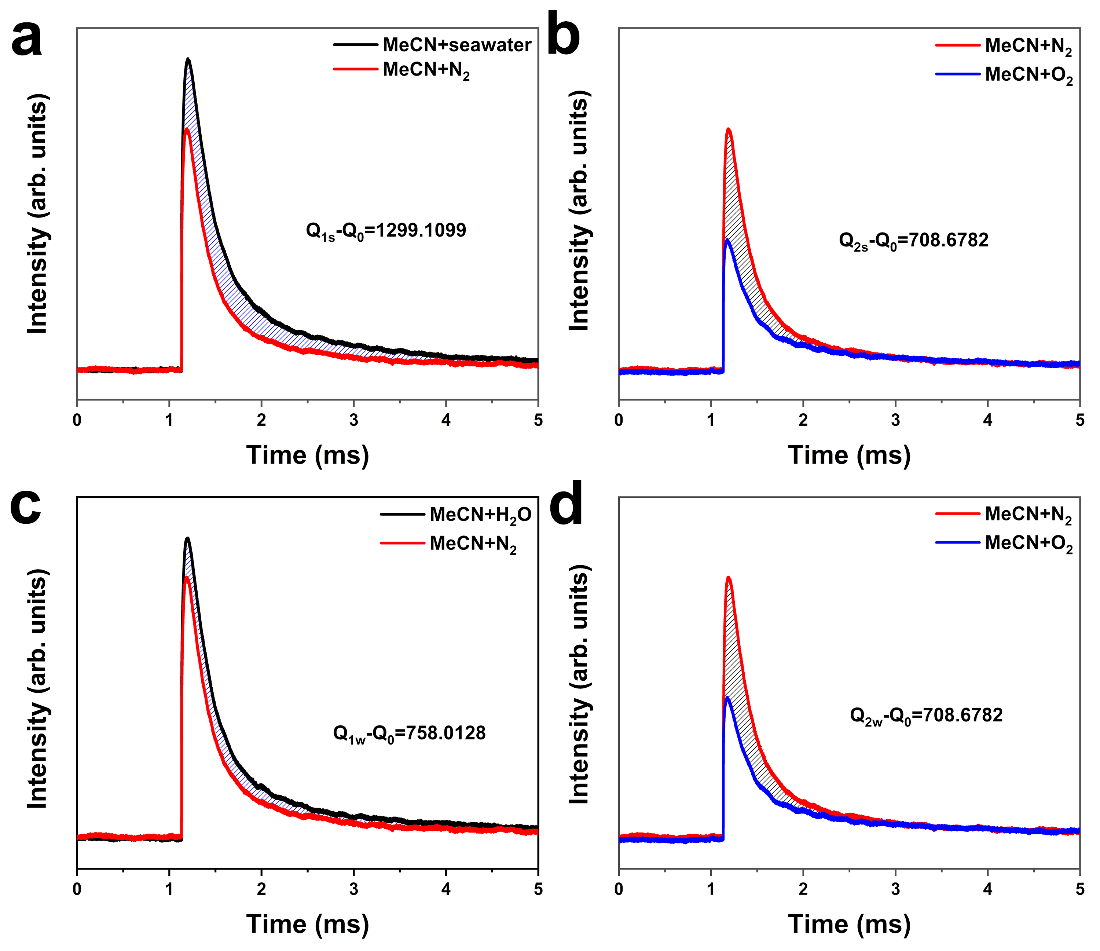


**Figure S15.** *In-situ* TPV spectra and integration of PM-CDs-30 under different conditions. (a) TPV curves and integration under N_2_-saturated acetonitrile and 0.1 vol% seawater/acetonitrile. (b) TPV curves and integration under N_2_-saturated acetonitrile and O_2_-saturated acetonitrile. (c) TPV curves and integration under N_2_-saturated acetonitrile and 0.1 vol% water/acetonitrile. (d) TPV curves and integration under N_2_-saturated acetonitrile and O_2_-saturated acetonitrile. Source data are provided as a Source Data file.


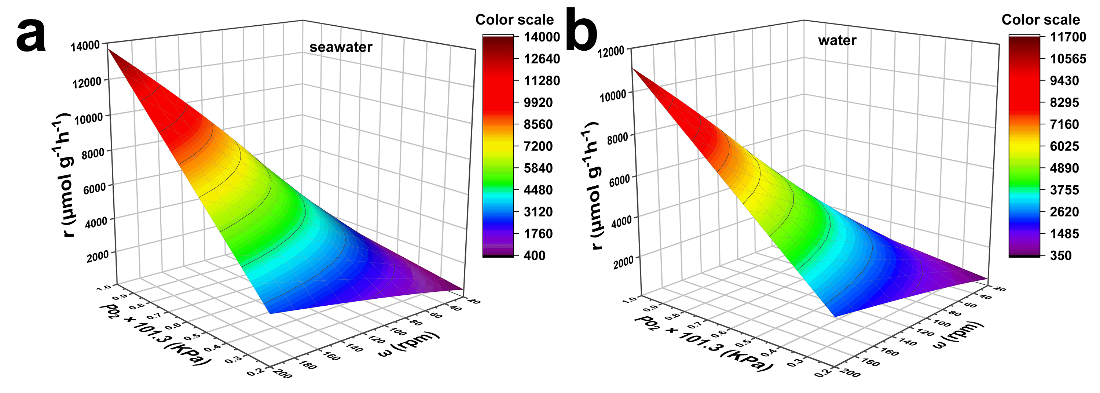


**Figure S16.** (a) Lattice diagram of rate for PM-CDs-30 freely varying with rotational speed and oxygen partial pressure in seawater. (b) Lattice diagram of rate for PM-CDs-30 freely varying with rotational speed and oxygen partial pressure in water.


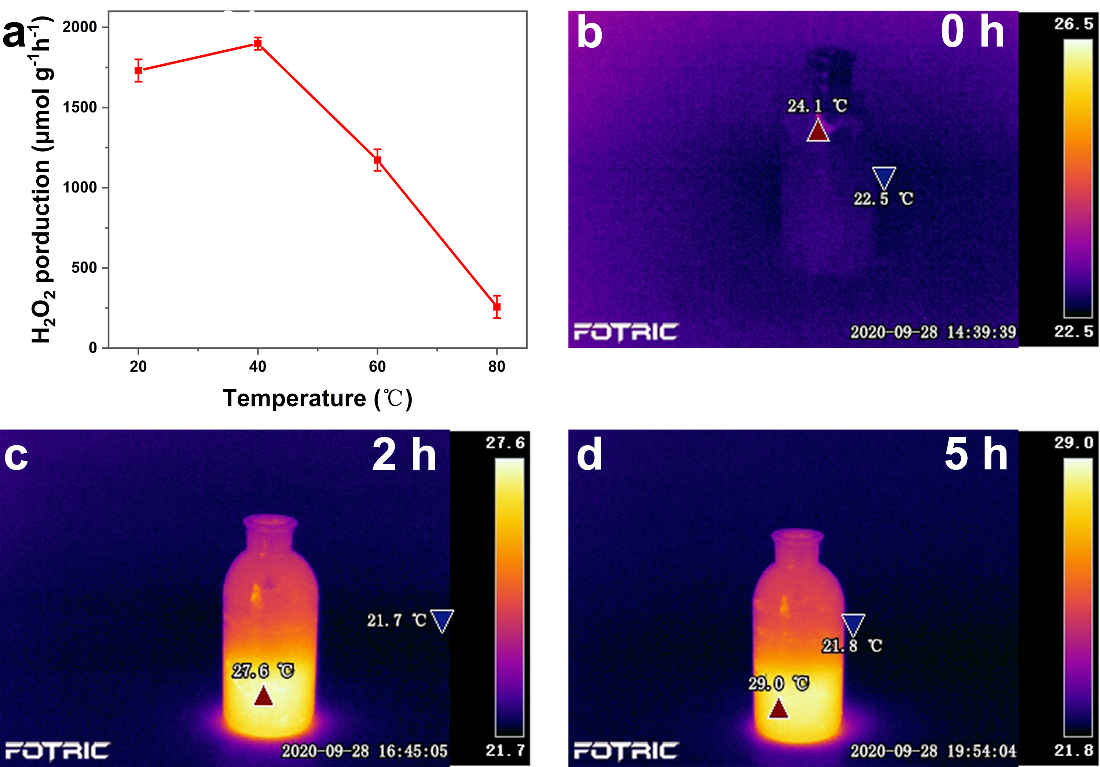


**Figure S17.** (a) Temperature course of H_2_O_2_ photoproduction by PM-CDs-30. (b) Infrared temperature maps of the reaction system before catalytic reaction at room temperature. The vertical error bars indicate the maximum and minimum values obtained; the square represents the average value. (c) Infrared temperature maps of the reaction system after catalytic reaction at room temperature for 2 h. (d) Infrared temperature maps of the reaction system after catalytic reaction at room temperature for 5 h. Source data are provided as a Source Data file.


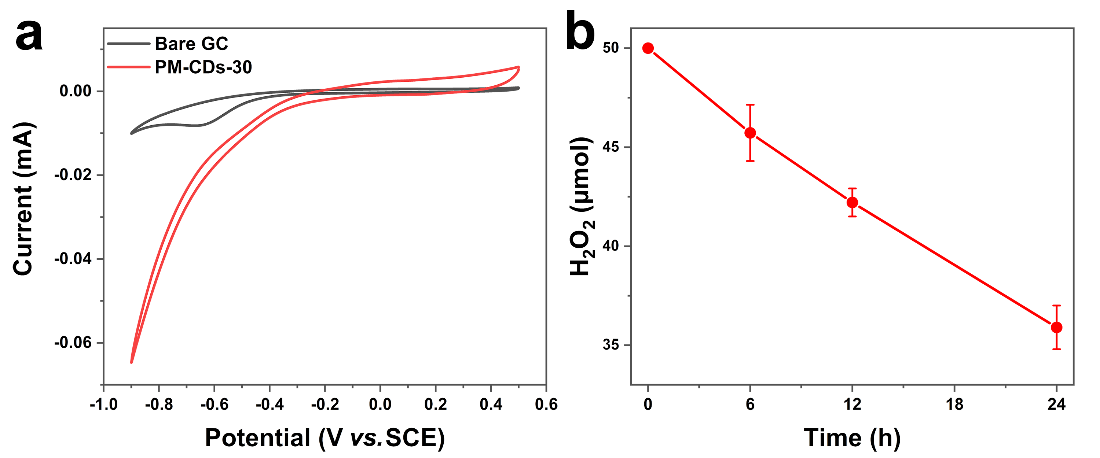


**Figure S18.** (a) Cyclic voltammograms curves of bare GC and PM-CDs-30 modified GC electrodes in 0.2 M (pH = 7) phosphate buffered 25 mM H_2_O_2_ solution at a scan rate of 50 mV s^-1^ (without light). (b) Change of H_2_O_2_ content in solution over time under dark condition. The vertical error bars indicate the maximum and minimum values obtained; the dot represents the average value. Source data are provided as a Source Data file.


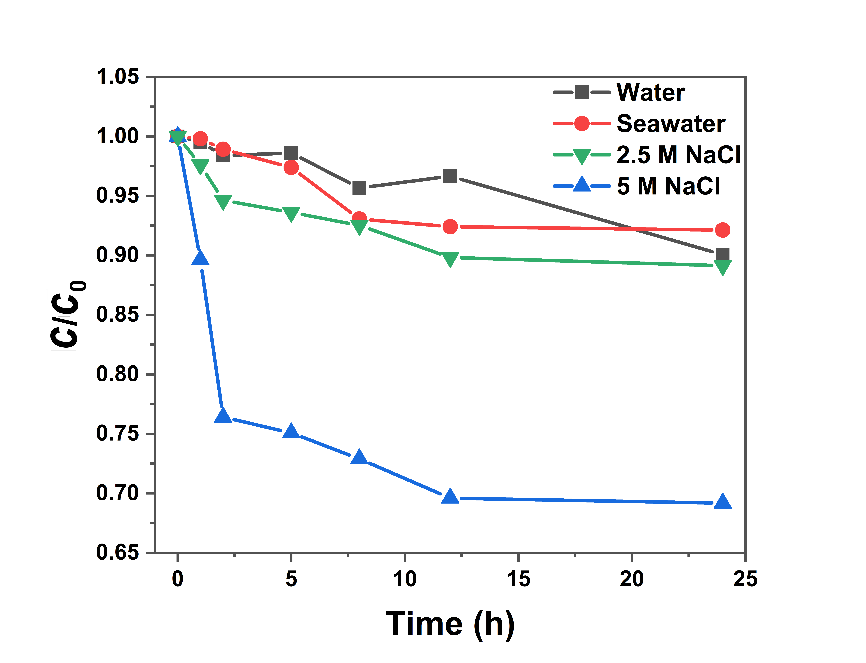


**Figure S19.** Changes in catalyst (PM-CDs-30) dispersion concentration over time in different solutions. Source data are provided as a Source Data file.


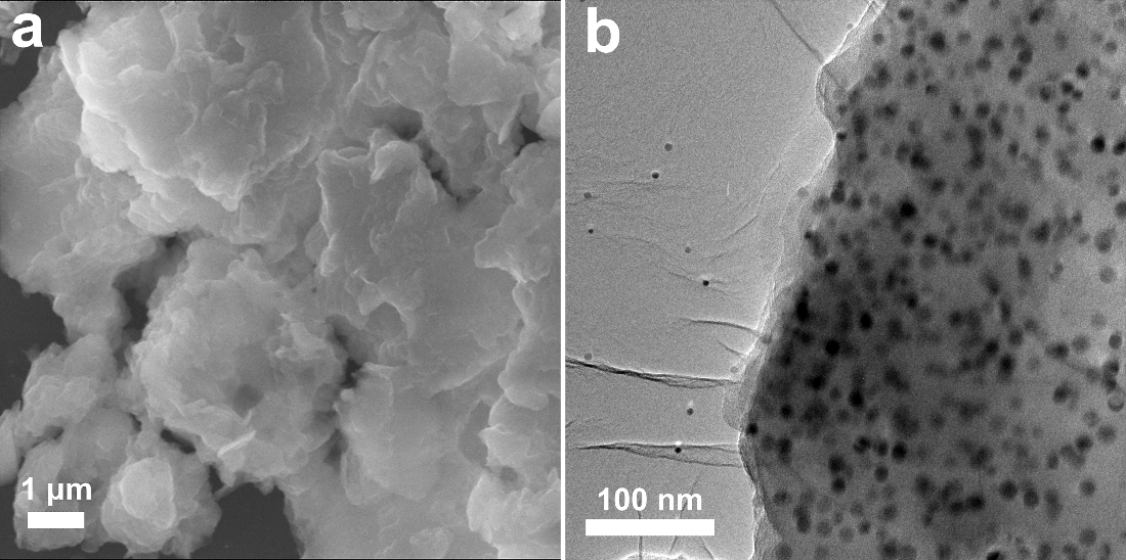


**Figure S20.** (a) SEM image of PM-CDs-30 after stability tests. (b) TEM image of PM-CDs-30 after stability tests.


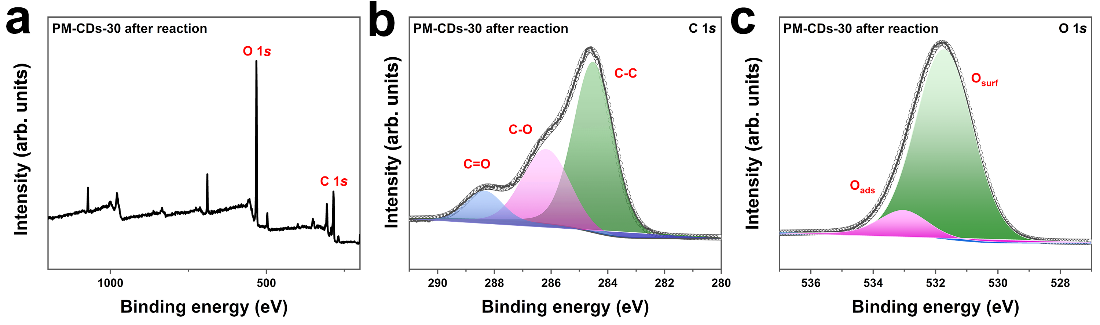


**Figure S21.** XPS spectra of PM-CDs-30 after stability tests. (a) Full spectrum. (b) C 1*s* spectrum. (b) O 1*s* spectrum. Source data are provided as a Source Data file.


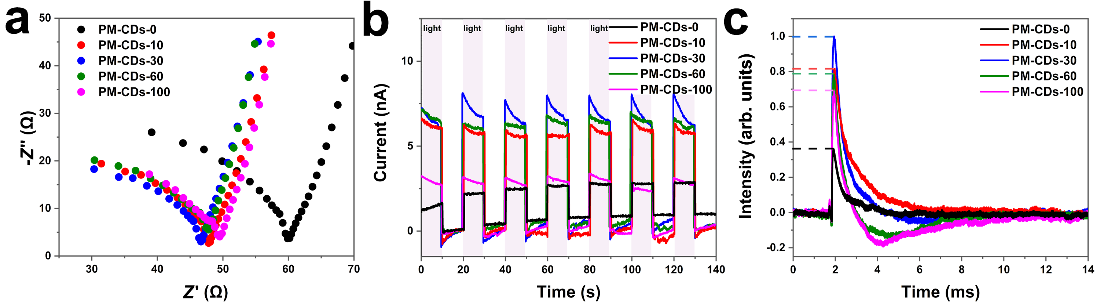


**Figure S22.** Photoelectrochemical tests of PM-CDs-x. (a) EIS Nyquist Plots in seawater. (b) Photo-response curves in seawater. (c)TPV patterns. Source data are provided as a Source Data file.


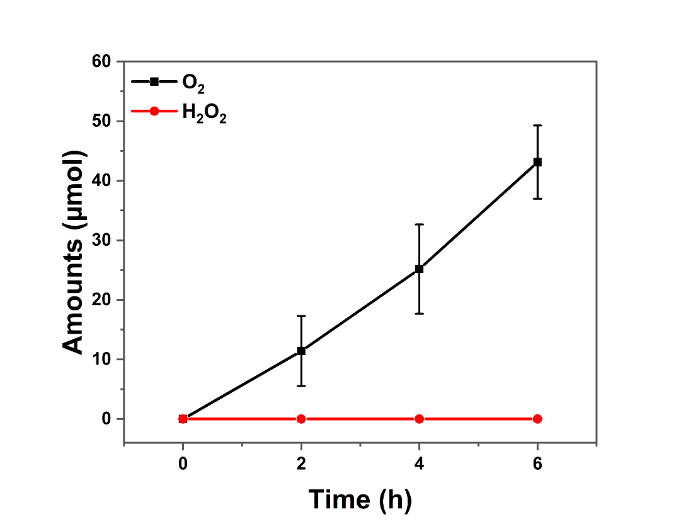


**Figure S23.** Amounts of O_2_ and H_2_O_2_ formed during the half photoreaction. Considerations: water (20 mL), PM-CDs-30 catalyst (10 mg), AgNO_3_ (10 mM), N_2_. The vertical error bars indicate the maximum and minimum values obtained; the dot and square represent the average value. Source data are provided as a Source Data file.


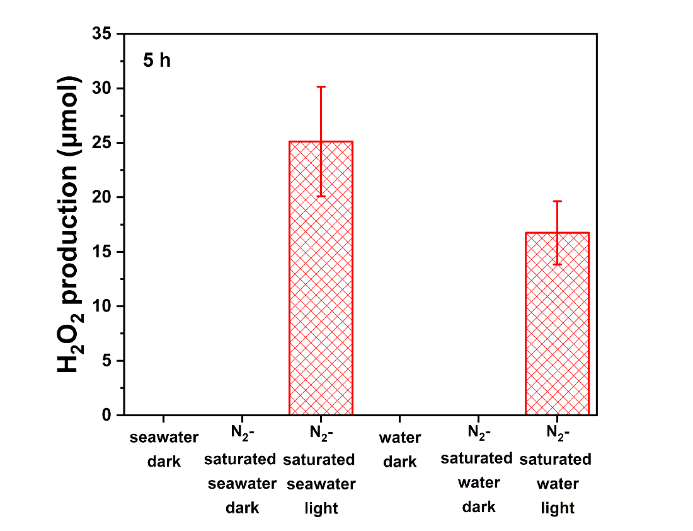


**Figure S24.** The production of H_2_O_2_ by photocatalytic reaction in different conditions. The vertical error bars indicate the maximum and minimum values obtained. Source data are provided as a Source Data file.


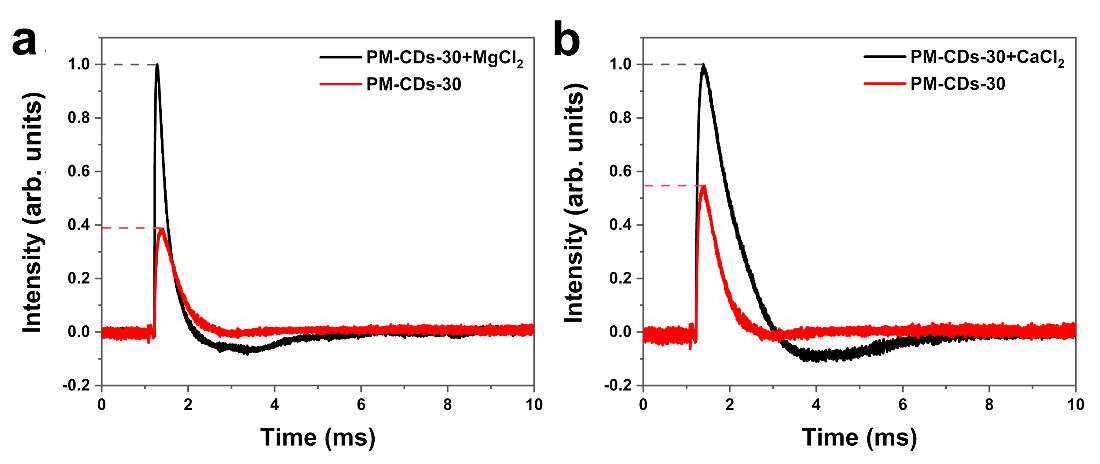


**Figure S25.** (a) TPV curves of PM-CDs-30 before and after adding MgCl_2_. (b) TPV curves of PM-CDs-30 before and after adding CaCl_2_. Source data are provided as a Source Data file.


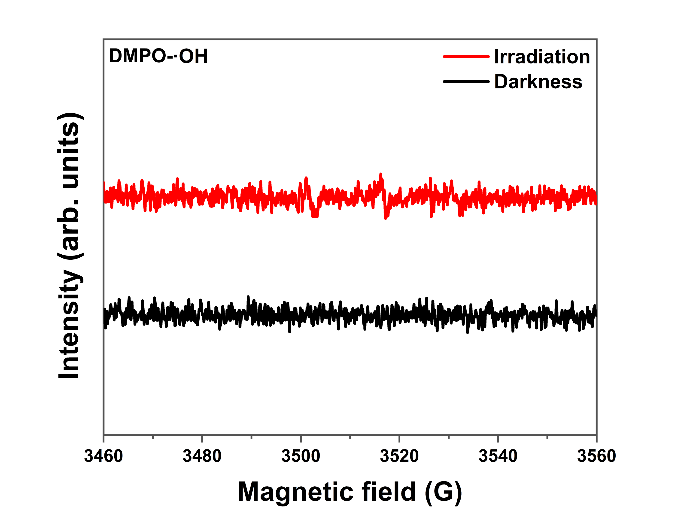


**Figure S26.** EPR spectra of PM-CDs-30 under darkness and light to detect hydroxyl radical. Source data are provided as a Source Data file.


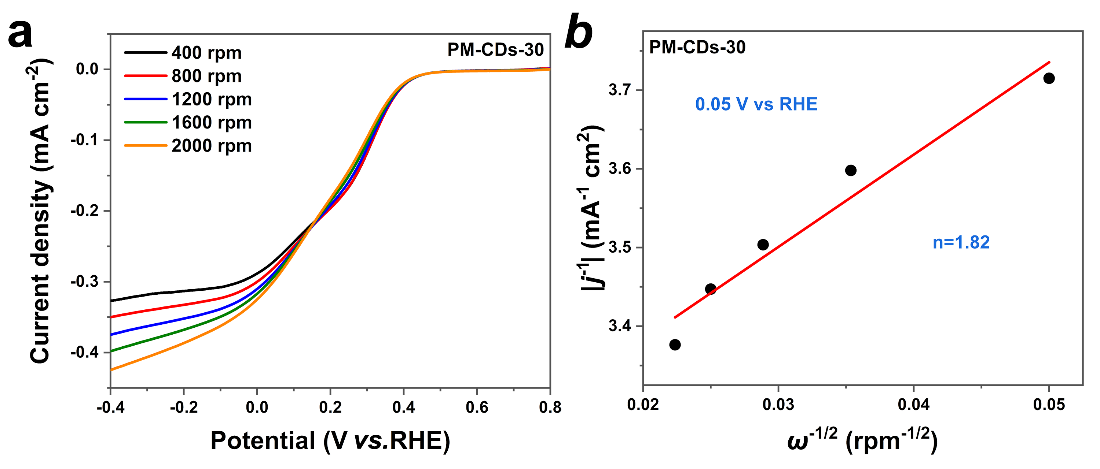


**Figure S27.** (a) LSV curves of PM-CDs-30 toward ORR in O_2_-saturation seawater with different rotating speeds. (b) K-L plots at 0.05 V (*vs.* RHE) and the electron transfer number under corresponding voltages of PM-CDs-30. Source data are provided as a Source Data file.


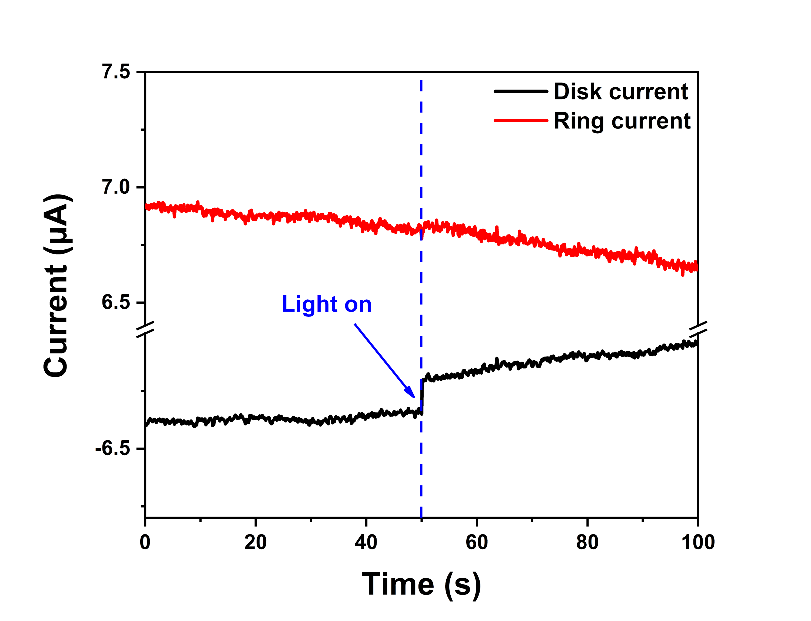


**Figure S28.** RRDE collection experiment of PC–MB-3 in N_2_-saturation seawater. Source data are provided as a Source Data file.

**5. Supplemental Table**

**Table S1.** Elemental compositions of PM-CDs-x samples from XPS and elemental analysis.

| **Photocatalyst** | **XPS**  **[at %]** | | **EA**  **[%]** | | | |
| --- | --- | --- | --- | --- | --- | --- |
|  | C | O | C | H | O | N |
| PM-CDs-0 | 72.42 | 27.58 | 63.96 | 5.01 | 30.9 | 0.13 |
| PM-CDs-10 | 72.95 | 27.05 | 62.48 | 5.13 | 32.24 | 0.15 |
| PM-CDs-30 | 73.4 | 26.6 | 62.72 | 5 | 32.12 | 0.16 |
| PM-CDs-60 | 73.59 | 26.41 | 62.58 | 5.03 | 32.2 | 0.19 |
| PM-CDs-100 | 73.79 | 26.21 | 63.55 | 4.96 | 31.25 | 0.24 |

^a)^ The element N in the samples may be derived from the residual HNO_3_. Moreover, the measurement error of the instrument is another reason for the appearance of the N element.

**Table S2.** The specific surface area of PM-CDs-x samples.

| **Photocatalyst** | **Specific surface area**  **[m^2^ g^-1^]** |
| --- | --- |
| PM-CDs-0 | 1.5076 |
| PM-CDs-10 | 4.9504 |
| PM-CDs-30 | 4.9129 |
| PM-CDs-60 | 5.1849 |
| PM-CDs-100 | 4.5879 |

**Table S3.** The intensity of light at different wavelengths.

| **Wavelength**  **[nm]** | 365 | 420 | 535 | 630 |
| --- | --- | --- | --- | --- |
| **Light intensity**  **[mV cm^-2^]** | 39.42 | 31.74 | 8.82 | 21.90 |

**Table S4.** Comparison of the catalytic activities of different photocatalyst systems in the literatures.

| Photocatalyst | Condition | H_2_O_2_  μmol/h | AQY at 420 nm | SCC  % | Ref. |
| --- | --- | --- | --- | --- | --- |
| **PM-CDs-30** | **Real seawater** | **17.76** | **0.99%** | **0.21** | **This work** |
| m-WO_3_/FTO-Co^II^(Ch)/CP cathode | O_2_-saturated [artificial](javascript:;) [seawater](javascript:;)  HClO_4_ (pH=1.3) and 0.1 M NaClO_4_ | 16 | - | 0.55 | 5 |
| TiO_2_ | 4% NaCl solution | 18 | - | - | 6 |
| FeO(OH)/BiVO_4_/FTO photoanode-Co^II^(Ch)/carbon paper cathode | O_2_-saturated [artificial](javascript:;) [seawater](javascript:;)  HClO_4_ (pH=1.3) and 0.1 M NaClO_4_ | 34 | - | 0.89 | 7 |
| Au/BiVO_4_ | O_2_-saturated water | 0.121 | 0.24% | - | 8 |
| g-C_3_N_4_/NaBH_4_ | Water | 17 | 4.3% | 0.26 | 9 |
| g-C_3_N_4_/BDI_50_ | O_2_-saturated water | 0.854 | 2.6% | 0.13 | 10 |
| g-C_3_N_4_/PDI/rGO | O_2_-saturated water | 1.21 | 6.1% | 0.2 | 11 |
| RF523 | O_2_-saturated water | 2.58 | 6% | 0.5 | 12 |

**Table S5.** The carbon and oxygen functionalities concentrations in C 1*s* spectra.

|  | **Before reaction**  **%** | **After reaction**  **%** |
| --- | --- | --- |
| C-C | 60.6 | 61.6 |
| C-O/C=O | 39.4 | 38.4 |

**6. Supplemental References**

1. Zhao, S. & Zhao, X. Polyoxometalates-derived metal oxides incorporated into graphitic carbon nitride framework for photocatalytic hydrogen peroxide production under visible light. *J. Catal.* **366**, 98–106 (2018).
2. Fu, Y. J. et al. Photocatalytic H_2_O_2_ and H_­2_ generation from living chlorella vulgaris and carbon micro particle comodified g-C_3_N_4_. *Adv. Energy Mater.* **8**, 1802525 (2018).
3. Kim, H. Choi, Y. Hu, S. Choi, W. & Kim, J. H. Photocatalytic hydrogen peroxide production by anthraquinone-augmented polymeric carbon nitride. *Appl. Catal. B-Environm.* **229**, 121-129 (2018).
4. Wang, G. C. et al. Modulating location of single copper atoms in polymeric carbon nitride for enhanced photoredox catalysis. *ACS Catal.* **10**, 5715−5722 (2020).
5. Mase, K., Yoneda, M., Yamada, Y. & Fukuzumi, S. Seawater usable for production and consumption of hydrogen peroxide as a solar fuel. *Nat. Commun.* **7**, 11470 (2016).
6. Harada, H. Isolation of hydrogen from water and/or artificial seawater by sonophotocatalysis using alternating irradiation method. *Int. J. Hydrogen Energ.* **26**, 303-307 (2001).
7. Mase, K., Yoneda, M., Yamada, Y. & Fukuzumi, S. Efficient photocatalytic production of hydrogen peroxide from water and dioxygen with bismuth vanadate and a cobalt(II) chlorin complex. *ACS Energy Lett.* **1**, 913–919 (2016).
8. Hirakawa, H. *et al.* Au nanoparticles supported on BiVO_4_: effective inorganic photocatalysts for H_2_O_2_ production from water and O_2_ under visible light. *ACS Catal.* **6**, 4976–4982 (2016).
9. Zhu, Z., Pan, H., Murugananthan, M., Gong, J. & Zhang, Y. Visible light-driven photocatalytically active g-C_3_N_4_ material for enhanced generation of H_2_O_2_. *Appl. Catal. B-Environ.* **232**, 19–25 (2018).
10. Kofuji, Y. *et al.* Graphitic carbon nitride doped with biphenyl diimide: efficient photocatalyst for hydrogen peroxide production from water and molecular oxygen by sunlight. *ACS Catal.* **6**, 7021–7029 (2016).
11. Kofuji, Y. *et al.* Carbon nitride–aromatic diimide–graphene nanohybrids: metal-free photocatalysts for solar-to-hydrogen peroxide energy conversion with 0.2% Efficiency. *J. Am. Chem. Soc.* **138**, 10019–10025 (2016).
12. Shiraishi, Y. *et al.* Resorcinol–formaldehyde resins as metal-free semiconductor photocatalysts for solar-to-hydrogen peroxide energy conversion. *Nat. Mater.* **18**, 985–993 (2019).
